# Relationship Status, Social Interactions, and Conversations in Late Life

**DOI:** 10.1093/geroni/igaa057.1336

**Published:** 2020-12-16

**Authors:** Yee To Ng, Meng Huo, Shiyang Zhang, Karen Fingerman

**Affiliations:** 1 The University of Texas at Austin, Austin, Texas, United States; 2 University of California, Davis, Davis, California, United States

## Abstract

Studies suggest spending more time interacting with and talking to others is associated with better well-being. Older adults with partners (e.g., married, cohabitated) may spend more time with their romantic partners and rely on them for support, whereas older adults without partners (e.g., widowed, divorced, never married) may have a greater reliance on other family members (e.g., grown children, siblings) and non-kin (e.g., friends). Yet, we know little about how older adults’ relationship status affects their time spending alone or with other social partners, and the frequency of conversation throughout the day. Adults aged 65+ (N = 313) completed an interview about their relationship status and social partners. They then reported social encounters in ecological momentary assessments every 3 hours for 5 to 6 days. Participants also wore Electronically Activated Recorders which captured snippets of their conversation throughout the day. Older adults with partners reported 85% of time was with their romantic partners. Multilevel models revealed that compared to older adults with partners, older adults without partners were more likely to spend time alone and have encounters with friends throughout the day. Older adults without partners also engaged in fewer conversations throughout the day. Further, older adults without partners talked significantly more when they encountered friends than did older adults with partners. Findings suggest that friends are important in older adults’ social networks particularly for those who do not have romantic partners. Daily contact with social partners facilitates conversations and that could have implications for emotional or cognitive health.

